# Comparative Transcriptome Analysis of *Thitarodes Armoricanus* in Response to the Entomopathogenic Fungi *Paecilomyces Hepiali* and *Ophiocordyceps Sinensis*

**DOI:** 10.3390/insects11010004

**Published:** 2019-12-19

**Authors:** Zhongchen Rao, Li Cao, Hua Wu, Xuehong Qiu, Guiqing Liu, Richou Han

**Affiliations:** Guangdong Public Laboratory of Wild Animal Conservation and Utilization, Guangdong Key Laboratory of Animal Protection and Resource Utilization, Guangdong Institute of Applied Biological Resources, Guangzhou 510260, China; xipredus@163.com (Z.R.); caol@giabr.gd.cn (L.C.); xhqiu@126.com (X.Q.); Pepsiliu81@163.com (G.L.)

**Keywords:** *Thitarodes armoricanus*, *Ophiocordyceps sinensis*, *Paecilomyces hepiali*, infection, transcriptome, immunity

## Abstract

*Thitarodes armoricanus* is a medicinal and economically important Lepidopteran insect species. The larvae infected by *Paecilomyces hepiali* survive no more than four days, while those infected by *Ophiocordyceps sinensis* can survive for several months before mummification. This provides a valuable comparative system to study interactions between an insect host and different pathogenic fungi. By using the *T. armoricanus* genome, a time-course transcriptome analysis of the whole larvae without guts was performed to explore the larvae response to *P. hepiali* and *O. sinensis infection*. A total of 3106 differentially expressed genes in five clusters were identified. The genes involved in coagulation and multiple metabolisms were both suppressed after *P. hepiali* or *O. sinensis* infection, whereas those related to environmental information responses, cell processes, biotic stimulus, and immunity (such as *cecropin* (*CEC*)) were elevated. The rapid death of *T. armoricanus* after *P. hepiali* infection might be caused by osmotic imbalance, immunocompromise (such as *DEFs* and *GLVs*), and nervous system dysfunction (glutamatergic synapse). Up-regulation of the genes related to cuticle structure, nervous system (such as neurotrophin signal pathway and dopaminergic synapse) and immune effectors (such as *attacin* (*ATT*) and *proline-rich antimicrobial peptide 1* (*PRAMP1*)) in *T. armoricanus*, may contribute to the co-existence of *T. armoricanus* and *O. sinensis*. This study provides a global view and potential key genes of the interaction between *T. armoricanus* and two fungal entomopathogens.

## 1. Introduction

The ghost moth, genus *Thitarodes* (Lepidoptera: Hepialidae), is distributed naturally on the Tibetan Plateau and its surrounding region at high altitudes of between 3600 and 4800 m. More than 40 different species of *Thitarodes* have been reported, because of the special geographic feature of its habitat and its limited migration ability [1]. In nature, the ghost moths require more than three years to complete the life cycle, including four developmental stages: egg, 1st–9th instars larva, pupa, and adults [2]. Interestingly, the 7th to 9th instar larvae could enter the pupa stage [3]. 

The larval stage holds most of the time feeding on roots underground, during this stage, the insects encounter different pathogens such as fungi, bacteria, and nematodes, as well as predatory mites and some small insects [4]. Especially, larvae of *Thitarodes* are host of the entomopathogenic fungus *Ophiocordyceps sinensis*. The fungus-caterpillar complex named Chinese cordyceps, which is one of the most valued tonic food and traditional Asian medicines with broad medical effects such as immunoregulation, antioxidation, and anti-atherosclerosis [2,5,6,7,8,9,10,11]. Due to its extensive medical use but limited distribution and over-exploitation [12,13], the annual yield have been reduced and the market price is increased rapidly [8]. Its price increases by 20% per year on average from 1997, and more than $83,300 per kilogram for high-quality products are indicated from Tongrentang Pharmaceutical Co. in Beijing on 11 April, 2019. The Chinese cordyceps has been listed as an endangered species (CITES-II) for protection (CITES Management Authority of China and China Customers 2000). During *T. armoricanus* rearing, *Paecilomyces hepiali* is regarded as a seriously contaminative fungus because it can infect and kill the host larvae in several days [14]. However, the larvae infected by *O. sinensis* can survive for several months before mummification. Therefore, the response of ghost moth to *P. hepiali* or *O. sinensis* is a good model for studying molecular interactions between an insect host and entomopathogenic fungi, and the results would be useful for improving artificial cultivation of Chinese cordyceps.

Insects rely on physical barriers and the innate immune system to defend against microbes [15,16,17]. The innate immune system is based on two main components, cellular immunity and humoral immunity [18]. The fast-acting responses are largely mediated by hemocytes circulating in the hemolymph, including coagulation and melanization of foreign objects, phagocytosis of microbes, and encapsulation of parasites [18,19,20,21]. The slow response is induced over the course of several hours following a systemic infection and is regulated to combat specific pathogen classes [22]. This kind of immune response is composed of immune signaling pathways. Immune signal modulation and transduction pathways include Toll [23], immune deficiency (IMD) [24], janus kinase/signal transducer and activators of transcription (JAK/STAT) [25], and c-Jun N-terminal kinase (JNK) [26], which are triggered by recognition of pathogen-associated molecular patterns (PAMPs) on the microbial surface, resulting in the expression of immune response effectors such as antimicrobial peptides (AMPs) and *lysozyme* (*LYZ*). All of these reactions constitute a complex and interconnected network that protects insects from invasion by microbes. A total of 258 candidate immunity-related transcripts were identified in *Hepialus xiaojinensis* in response to *O. sinensis*, which is more primitive than other Lepidopteran insects, and *H. xiaojinensis* was capable of a rapid response to an *O. sinensis* challenge and developed tolerance to the fungus after prolonged infection by immune suppression [27]. Genes encoding physical barriers such as cuticle proteins, peritrophic matrix proteins, AMPs, pattern recognition receptors (PRRs), and enzymes in the proteolytic cascade were predicted to be involved in the response of *Thitarodes jiachaensis* to *O. sinensis* infection [28], and the gene families such as AMPs and *c-type lectins* (*CTLs*) in ghost moth have been well studied [29,30]. However, a global comparison of the ghost moth response to the challenge of *P. hepiali* and *O. sinensis* is still lacking. To study how the ghost moth response to these two fungi, high-throughput RNA sequencing (RNA-seq) was used to analyze time-course transcriptome profiles of *T. armoricanus* larvae infected with *P. hepiali* and *O. sinensis*. We identified the differentially expressed genes (DEGs) from larvae infected with *P. hepiali* or *O. sinensis* and proposed possible mechanisms of *T. armoricanus* responses to both fungi. The potential key genes for the interaction between the host insect and the two entomopathogenic fungi were also predicted. These results will facilitate the artificial production of this insect and the sustainable utilization of *O. sinensis*.

## 2. Materials and Methods 

### 2.1. Experimental Insects and Fungi

*T. armoricanus* pupae were collected from alpine meadows in Kangding, Sichuan, China. The larvae were reared on carrots in the laboratory at 9–13 °C in Guangzhou (43 m above sea level), Guangdong Province [3]. *P. hepiali* and *O. sinensis* were respectively isolated from the cadavers of *T. armoricanus* larvae. *O. sinensis* was cultured in PPDA liquid medium at 13 °C for three months and *P. hepiali* for 15 days. The spores from the two fungi were collected and in phosphate buffer saline (PBS) to a concentration of 10^9^ spores/mL. Healthy 7th instar larvae with body weights ranging from 0.5 g to 0.7 g for each larva was injected with 10 µL fungal suspension. The larvae injected with 10 µL 1 × PBS buffer were used as a control. Hemolymph from each larva was observed to confirm the presence of the infected spore under microcopy.

### 2.2. Preparation of RNA, Library Construction, and Sequencing

Guts were removed before extracting RNA, for guts of *T. armoricanus* contain too many microbes and made it difficult to assemble. We dissected the larvae in PBS, guts were pull out by using forceps. larvae without gut were immediately frozen in liquid nitrogen and store at −80 °C. Total RNA was extracted with TRIzol according to the manufacturer’s instruction. A Nanodrop ND-2000 spectrophotometer, non-denaturing agarose gel electrophoresis, Qubit 2.0 and Agilent 2100 Bioanalyzer (Agilent, Santa Clara, CA, USA) were used to determine the quantity and quality of RNA in the samples. A total of 15 individual cDNA libraries were constructed from the larvae of three days (PH3d) post *P. hepiali* injection, three days (OS3d) and 15 days (OS15d) post *O. sinensis* injection, and three days (PBS3d) and 15 days (PBS15d) post PBS-injection. Each treatment with three biological replicates, and 5–6 larvae were used for each biological replicate. The quantification and qualification of the libraries was analyzed on Qubit 2.0, Agilent 2100 Bioanalyzer (Agilent, Santa Clara, CA, USA) and ABI StepOnePlus Real-Time PCR system (ABI, Waltham, MA, USA). An Illumina NovaSeq 6000 platform (Illumina, San Diego, CA, USA) was used for sequencing. All of the raw sequence data were deposited in the NCBI Sequence Read Archive (SRA) database under BioProject accession No. PRJNA580356.

### 2.3. Mapping and Transcriptome Annotation

The reads that contained adapter sequences, with more than 10% uncertain base pairs, and with low quality were removed. The resulting clean reads were used to perform quality control through base composition and quality distribution. Only the clean reads with a balanced composition, as well as high distribution of high-quality base (sequencing quality value > 20) were kept. The remaining clean reads were mapped to the genome of *T. armoricanus* [31] using HISAT2 (2.0.6) [32]. StringTie (v1.0.4) was used to reconstruct transcripts [33], and the potential novel transcripts were predicted by cufflinks (v2.2.1) [34]. All of the novel transcripts were annotated against the NCBI non-redundant protein database and Swiss-prot database using BlastX (E-value < 1 × 10^−5^).

### 2.4. Differentially Expressed Gene Analysis

RNA-seq by expectation maximization (RSEM, v 1.2.31) [35], a utility package in the software Trinity, was used to estimate the abundance of transcripts and the fragments per kilobase per million mapped read (FPKM), which were calculated for the digital gene expression profile. Differentially expressed genes (DEGs) were calculated using edgeR [36]. *p*-values were corrected for multiple hypothesis tests, and the threshold *p*-value by False Discovery Rate (FDR) was determined. Genes in different samples with FDR < 0.05 and |fold change| > 2 were considered as DEGs. 

### 2.5. Clustering and Enrichment Analysis

Clustering analysis for DEGs in *T. armoricanus* was performed using Cluster3.0 [37] based on the Euclidean distance. A strict algorithm was developed to carry out enrichment analysis for the gene list of DEGs in *T. armoricanus* based on the algorithm presented by GO::TermFinder, *p*-values were Bonferroni [38] corrected, and the threshold for corrected *p*-values was set to ≤0.05. The GO enrichment comparison between different clusters was carried out using WEGO [39]. The pathway enrichment analysis of DEGs was performed with KOBAS 3.0 [40] with a threshold *p*-value set to ≤0.05. The unit numbers in the enriched pathway of different clusters were assigned to KEGG (Kyoto Encyclopedia of Genes and Genomes) mapper [41].

### 2.6. Identification of Immunity or Defense Related Genes from T. armoricanus

The immunity or defense related genes from different species were obtained from following three ways: (1) ImmunoDB (http://cegg.unige.ch/Insecta/immunodb); (2) References; (3) Genes with GO annotation that related to immune or defense responses (https://www.ebi.ac.uk/QuickGO/), The GO terms for use were shown in Table S1. These genes were used as references for Blast search against *T. armoricanus* genome (identified > 30%, coverage > 50%), these potential immunity or defense related genes were then filtered by Blast search against NR and Swiss-Prot databases, and confirm manually. Domain prediction was performed by Pfam (v30.0), CDD (v3.16) and SMART (v6.0). The putative signal peptide and transmembrane helice were predicted by SignalP (v4.1) and TMHMM (v2.0), respectively. The immunity or defense related genes were classified into several group: fast-acting response to foreign bodies (melanization and coagulation), microbes (phagocytosis) and parasites (encapsulation) [22]; slow response after several hours following a systemic infection (Toll, IMD, JNK and JAK/STAT); self-devouring (autophagy and apoptosis); defense from oxidative stress (removal of superoxide radicals); virus defense (small regulatory RNA pathway); genes involved in immune or defense response but not classified in the above groups (others).

### 2.7. Sequence Analysis and Phylogenetic Analysis

The protein sequences of Clip-domain serine proteases (cSPs) and Clip-domain serine protease homologs (cSPHs) in *T. armoricanus* and *Plutella xylostella*, and the Gastrulation-defectives (*GD*) in different species, were identified from the corresponding genomes. Subfamilies of cSPs and cSPHs families were well studied in *Drosophila melanogaster*. Gene list of cSPs and cSPHs in *D. melanogaster* was obtained from previous review [42], and the sequences were extracted from flybase (v2017_06, http://flybase.org/). The references protein sequences (in silico translated) for Blast search were obtained from ImmunoDB and Swissprot (https://www.uniprot.org/uniprot/?query=reviewed:yes). The BlastP (v2.2.31) was used to compare the sequences in the database. The methods for predicting domains, signal peptides, and transmembrane structure were done as described above. The Cluster3.0 [37] multiple sequence alignment program was used to align the sequences of cSPs and cSPHs. The phylogenetic tree was constructed by MEGA 6.06 program [43], and visualized by Evolview [44].

### 2.8. Quantitative Real-Time PCR

RNA from *T. armoricanus* larvae injected with *P. hepiali*, *O. sinensis* and PBS for three days and 15 days were used to validate the RNA-seq experiment. A total of 1 μg of RNA from the transcriptome sample was used for cDNA synthesis according to the manufacturer’s protocol (PrimeScript™ RT Reagent Kit with gDNA Eraser, TaKaRa, Japan). The 25-μL reaction consisted of 2 μL of diluted cDNA (1:2), 12.5 μL of SYBR^®^ Premix Ex Taq II (Tli RNaseH Plus) (TaKaRa, Kyoto, Japan) and 0.2 mM of each primer, were used for qPCR reaction. All reactions were performed on a Stratagene MX3000P qPCR system (Stratagene, Santa Clara, CA, USA), according to the manufacturer’s instructions. Thermal cycling conditions were set to 95 °C for 1 min of initial denaturation, followed by 40 cycles of 95 °C for 15 s, 60 °C for 30 s, and 72 °C for 30 s of amplification. Then, a melting curve analysis from 55 °C to 95 °C was applied to all reactions to ensure consistency and specificity of the amplified product. All primers used for the genes of interest are described in Table S2. Quantitative measurements were normalized by the reference gene ribosomal protein S3 and Glyceraldehyde-3-phosphate dehydrogenase, and relative expression levels were calculated using 2^−ΔΔCt^ method [45,46]. In the regression analysis, the fold changes of RNA-seq were base-2 logarithm of FPKM ratio, the fold changes of qPCR were ΔΔCt [45].

## 3. Results

### 3.1. RNA Sequencing Analysis 

Whole genome mRNA sequencing was used to monitor changes in gene expression among larvae after *P. hepiali* or *O. sinensis* infection. In total, 182.98 Gb high quality clean data was obtained from fifteen libraries, including nine infected samples (PH3d, OS3d, and OS15d) and six blank controls (PBS3d and PBS15d), with at least 54.87% clean reads matching to the *T. armoricanus* genome [31], using HISAT2 (Table S3). The FPKM value of expressed genes was shown in Table S4. The correlation coefficient between the biological replicated samples were shown in Figure S1a. The correlation coefficient between different samples of the same treatment always exceeded 0.95, and were higher than the values between the different treatment samples. A similar result was obtained in the principle components analysis (PCA) (Figure S1b).

### 3.2. Gene Responses to P. hepiali or O. sinensis Infection

**Figure 1:**
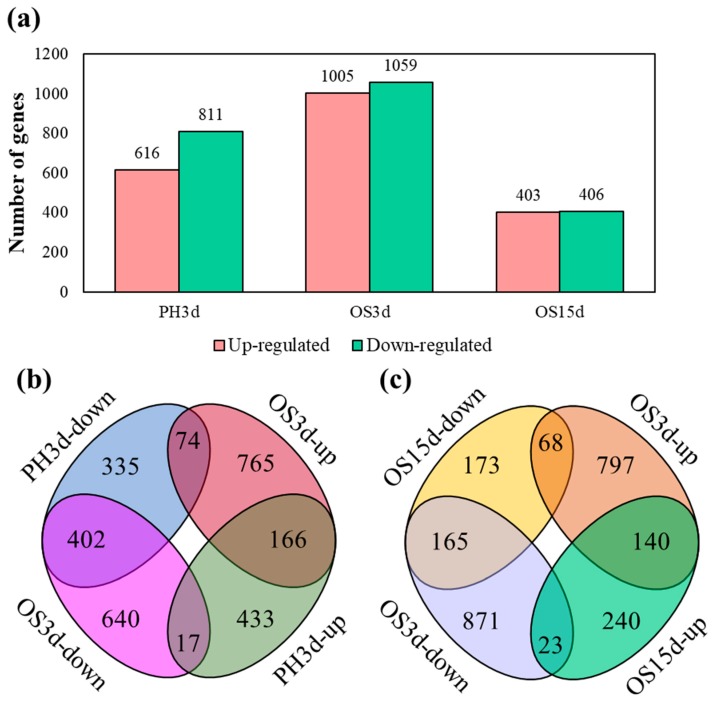
Differentially expressed genes (DEGs) of *T. armoricanus* larvae in response to *P. hepiali* or *O. sinensis* infection. (**a**) The number of up- and down-regulated genes in PH3d, OS3d, and OS15d. Venn diagram illustrating the common and specific genes of up- or down-regulated genes in (**b**) PH3d and OS3d, (**c**) OS3d and OS15d. PH3d—3 days post *P. hepiali* infection; OS3d—3 days post *O. sinensis* infection; OS15d—15 days post *O. sinensis* infection.

To gain insights into global transcriptional changes in *T. armoricanus* larvae infected by *P. hepiali* or *O. sinensis*, pairwise comparisons were performed between the *P. hepiali* or *O. sinensis* challenged libraries and the PBS-injected control to identify DEGs. A total of 3106 DEGs were obtained (FDR < 0.05 and |log_2_foldchange| > 1), including 1427, 2064, and 809 DEGs from PH3d, OS3d, and OS15d, respectively (Figure 1a). A complete list of significant DEGs is provided in Table S5. 

A similar number of up- and down-regulated genes were identified in OS3d and OS15d. Among these, the specifically up-regulated genes in OS3d (765), were 77% more numerous than those in PH3d (433). Specifically, 74 genes were up-regulated in OS3d but down-regulated in PH3d (Figure 1b). Notably, three days data probably showed an optimal expression from the insect in response to *P. hepiali* infection, thus no so many up-regulated genes were observed at this stage. A total of 807 DEGs were identified in OS15d, including 403 up-regulated and 406 down-regulated genes. When infected with *O. sinensis*, compared to three days after infection, 1668 DEGs (81% of DEGs in OS3d) restored to normal expression (FDR > 0.05 or |log2foldchange| < 1) after 15 days infection, including 797 up-regulated and 871 down-regulated genes in OS3d, while another 240 genes were specifically up-regulated in OS15d (Figure 1c). 

### 3.3. Transcriptional Changes During P. hepiali or O. sinensis infection 

Five major gene clusters were identified with distinct expression patterns based on the gene expression profiles in PH3d and OS3d by using Cluster3.0 (Table 1). 

Cluster 1 and Cluster 5 represented the common event that occur after three days post *P. hepialid* and *O. sinensis* infection. Cluster 1 including 402 genes, were down-regulated both in PH3d and OS3d. Genes related to digestion such as *alpha-amylase*, *trypsin*, *chymotrypsin,* and probable salivary secrete peptide were significantly down-regulated in the larvae in PH3d and OS3d (Table S5), indicating that the digestion relative genes were the commonly affected targets of fungi infection. Cluster 5 including 166 genes, were up-regulated both in PH3d and OS3d. Several DEGs with high expression, such as *Attacin* (*ATT*), *gloverin* (*GLV*), *lysozyme* (*LYZ*), *cecropin* (*CEC*), and defense protein, were related to immune response (Table S5).

Cluster 2 represented the genes specifically down-regulated after three days post *P. hepialid* or post *O. sinensis* infection. Cluster 2a included 409 genes, were specifically down-regulated in PH3d. three most significantly down-regulated genes were *L-ascorbate oxidase*, *osiris 9,* and *cuticle protein* (Table S5). Additionally, the immunity-related genes, such as *defensin* (*DEF*) and *proline-rich antimicrobial peptide 1* (*PRAMP1*) [47,48], were significantly down-regulated in PH3d (Table S5). Interestingly, these genes were up-regulated in OS3d and OS15d, indicating that *P. hepiali* (but not *O. sinensis*) might severely injure *T. armoricanus* by suppressing the expression of the set of specific immunity-related genes mentioned above. Cluster 2b included 657 genes, were specifically down-regulated in OS3d. Multiple genes related to fatty acid processes in Cluster 2b, such as *fatty acid desaturase* (*FAD*), *fatty acyl-CoA reductase* (*FAR*), *fatty acid synthase* (*FAS*), *Stearoyl-CoA desaturase*, *long-chain fatty acid transport protein 1* (*LFATP1*), *elongation of long chain fatty acids protein* (*ELOVL*) were down-regulated in OS3d (Table S5), implying fatty acid metabolism may significantly suppressed after *O. sinensis* infection (Table S5).

Cluster 3 represented the genes specifically differentially expressed after 15 days post *O. sinensis* infection. Totally 274 genes did not respond to PH3d or OS3d, but differentially expressed in OS15d, including 185 up-regulated genes (Cluster 3b) and 89 down-regulated genes (Cluster 3a). 

Cluster 4 represented the genes specifically up-regulated after three days post *P. hepialid* or post *O. sinensis* infection. Cluster 4a contained 433 genes, were specifically up-regulated in PH3d. Multiple pathogen or ligand receptors were well represented in this Cluster, such as *peptidoglycan recognition protein* (*PGRP*), *CTL*, *beta-1*,*3-glucan-binding protein* (*BGBP*) and *scavenger receptor class B member 1* (*SCARB1*), suggesting the important roles of these genes in *T. armoricanus* after *P. hepiali* infection. Cluster 4b contained almost two times more genes than Cluster 4a (765 vs. 433), indicating that *T. armoricanus* may be more sensitive to *O. sinensis* infection (Table S5). Especially, 93 cuticle related genes were specifically up-regulated after *O. sinensis* infection.

### 3.4. Gene Ontology (GO) and Pathway Analysis of Different Clusters in DEGs

**Figure 2:**
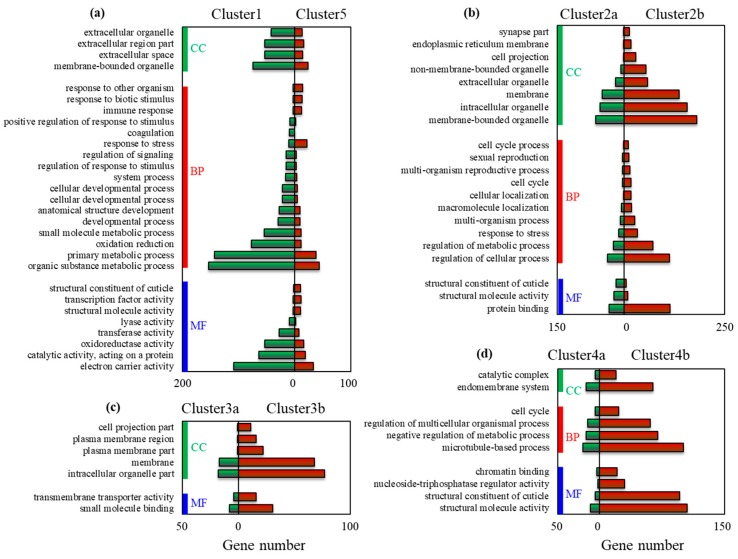
GO analysis of the DEGs in different clusters. The significantly differential GO terms between different clusters were shown, including (**a**) Clusters 1 and 5; (**b**) Cluster 2a,b; (**c**) Cluster 3a,b; and (**d**) Cluster 4a,b. Only when the ratios of gene number in different Clusters were more than 3, the corresponding GO terms were shown. Y-axle showed the GO terms, while the right and left part of X-axle represented the gene number of different clusters in corresponding GO terms (divided by “0”). CC—cellular component; BP—biological process; MF—molecular function.

The DEGs in different clusters were respectively used as inputs to perform GO annotation. For Cluster 1 vs. Cluster 5, 62 significantly differential (log_2_|cluster ratio| > 1, *p* < 0.05) GO terms were assigned to 355 genes (Table S6). Coagulation, oxidation reduction, lyase activity, electron carrier activity, and cofactor binding were well presented in Cluster 1, while response to biotic stimulus, immune response, response to other organism, were well represented in Cluster 5 (Figure 2a). For Cluster 2a vs. Cluster 2b, 46 significantly differential GO terms were assigned to 723 genes (Table S6), cuticle related genes were well represented in Cluster 2a, and the remaining were well represented in Cluster 2b, such as cell projection, membrane and organelle organization, localization, and binding. (Figure 2b). For Cluster 3a and Cluster 3b, 17 GO terms were significantly well presented at Cluster 3b. For Cluster 4a vs. Cluster 4b, 32 significantly differential GO terms were assigned to 795 genes (Table S6). Endomembrane system, microtubule-based process, structural molecule activity, structural molecule activity, and chromatin binding were significantly well represented in Cluster 4b, but no GO terms were well represented in Cluster 4a (Figure 2d).

**Figure 3:**
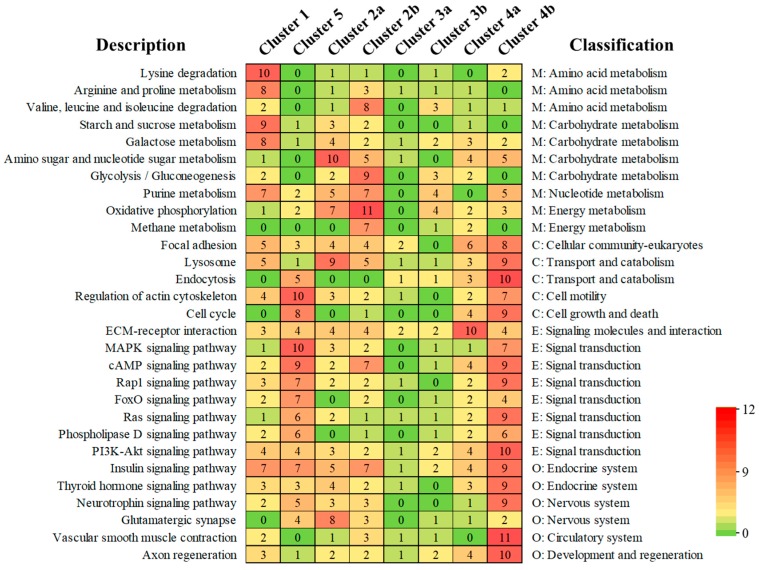
Heatmap for significantly differential pathways of different clusters in *T. armoricanus*. The pathway description (level 3) and classification (level 2) of pathways are shown, and 5 parent categories (level 1) were also defined: M—metabolism; E—environmental information processing; C—cellular processes; O—organismal systems. All of the pathways shown here represent at least one Cluster was enriched in this pathway (*p* < 0.05). The number in the box represents the functional unit (one unit may represent more than one gene) in corresponding pathways from different clusters.

In addition to the GO analysis, a KEGG enrichment was also implemented by analyzing the function of DEGs in the distinct clusters (Table S7). For Cluster 1 vs. Cluster 5, lysine degradation, arginine, proline, starch, sucrose, and galactose metabolism were well-represented in Cluster 1. Meanwhile, the signal transduction related to environmental information processing (such as MAPK, cAMP, Rap1, PoxO, and Ras signaling pathway), and cell processes (such as regulation of actin cytoskeleton and cell cycle) were well-represented in Cluster 5 (Figure 3). For Cluster 2a vs. Cluster 2b, amino and nucleotide sugar metabolism, cell processes (such as lysosome), and organismal systems (thermogenesis and glutamatergic synapse) were well-represented in Cluster 2a, while oxidative phosphorylation and glycolysis/gluconeogenesis was well-represented in Cluster 2b. For Cluster 4a vs. Cluster 4b, many pathways were well-represented in Cluster 4b, including pathways involved in environmental information processing (such as PI3K-Akt and Ras signaling pathways), cell processes (lysosome and endocytosis), and organismal systems (such as vascular smooth muscle contraction, axon regeneration, insulin and thyroid hormone signaling pathways). These annotations provide valuable information for further studying specific biological and metabolic processes, functions, and molecular mechanisms in *T. armoricanus*.

### 3.5. Expressed Changed from Three Days to 15 Days After O. sinensis Infection

In addition to the common changed between the OS3d/PBS3d and OS15d/PBS15d, we also measured the differential expression changed basing pairwise comparisons that performed between the three days and 15 days after *O. sinensis* challenged libraries (OS15d/OS3d) or PBS-injected libraries (PBS15d/PBS3d). Totally 1891 genes were differentially expressed, and 999 genes out of which were enhanced as the infection time increased. Pathway analysis show that these genes were enriched in multiple metabolism such as ascorbate, aldarate, methane, galactose, glycine, serine and threonine metabolism. Especially, 229 genes were differentially expressed after injected with PBS control, but expressed consistently after *O. sinensis* infection, suggesting the infection of *O. sinensis* may contribute to maintain these genes expression. Pathway analysis show that these genes were mainly enriched in fatty acid metabolism and fat digestion and absorption (Table S8). 

### 3.6. Immunity or Defense Related Pathways in T. armoricanus

To analyze the responses of *T. armoricanus* larvae after *P. hepiali* and *O. sinensis* infection, the immune or defense related genes were identified from the unpublished *T. armoricanus* genome (Table S9), and 292 genes in total were differentially expressed when the larvae were challenged with *P. hepiali* or *O. sinensis* infection (Table S10). The up-regulated genes from the larvae after three days post *P. hepiali* infection were more than those after three days post *O. sinensis* infection (66 vs. 43 genes), while the down-regulated genes after *P. hepiali* infection were fewer than those after *O. sinensis* infection (23 vs. 33 genes) (Table 2). Moreover, 53 genes were identified up-regulated at 15 d after *O. sinensis* infection (53), which were much more than those of down-regulated (18).

A total of 10 genes in coagulation, key genes phenoloxidase (*PO*) in melanization, caspase-1 in apoptosis pathway were significantly suppressed after both *P. hepiali* and *O. sinensis* infection (Table S10). Pathways involved in free radical and virus defending were also suppressed or did not activate after two fungi infection. While some other genes such as *calpain-A* (*CalpA*) and *THO complex subunit 1* (*THO1*) with antifungal activities [49,50], were specifically up-regulated after *O. sinensis* infection. These genes may be important to defend *O. sinensis* infection in *T. armoricanus* (Table S10).

**Figure 4:**
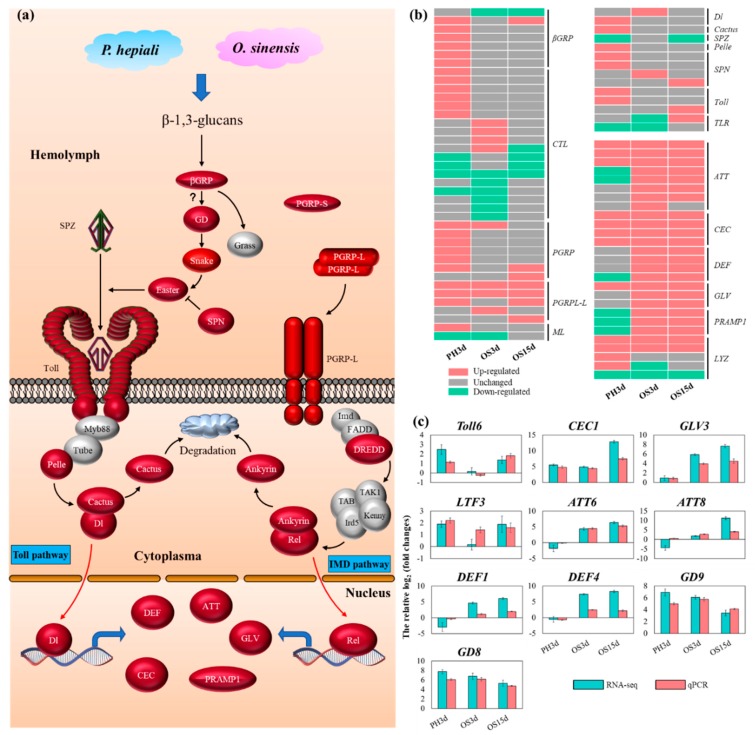
Toll and immune deficiency (IMD) pathways in response to *P. hepiali* and *O. sinensis* infection. (**a**) The potential Toll and IMD pathways in *T. armoricanus*. The genes differentially expressed or unchanged after *P. hepiali* or *O. sinensis* were shown in red and grey, respectively. (**b**) The relative log_2_foldchange of RNA-seq results of Toll and IMD pathway genes. The up-regulated, unchanged and down-regulated genes were shown by red, grey and green block, respectively. (**c**) qPCR results of 10 immune related genes. Green and red columns represented the RNA-seq and qPCR results, respectively.

The humoral immune response mainly contains four steps: pathogen recognition, signal modulation, intracellular signal transduction, and pathogen elimination by immune response effectors (Figure 4). Seventy PRRs were identified in *T. armoricanus* (Table S9), 39 of which were differentially expressed (Table S10).

**Figure 5:**
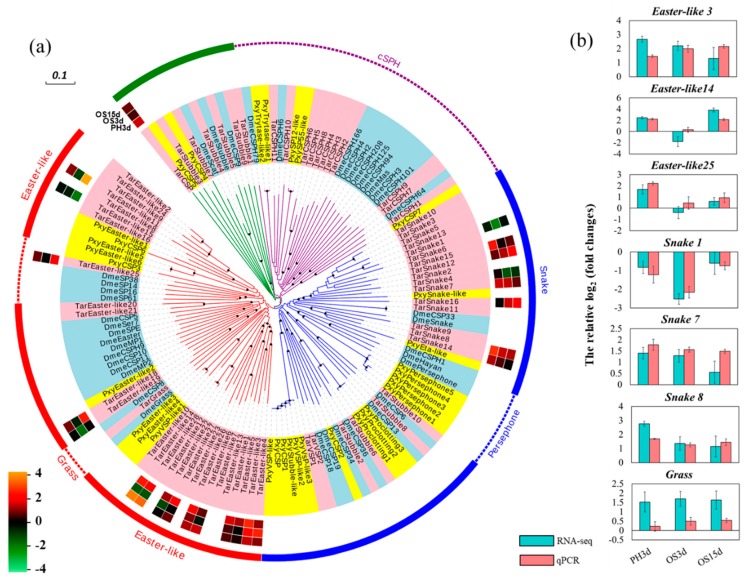
cSP family in *T. armoricanus*. (**a**) Phylogenetic analysis of cSP family. The method of neighbor joining (NJ) was applied for the construction of the phylogenetic tree with bootstrap value of 1000, and the bootstrap values >80 were shown on the tree with black dots. Four distinct gene clusters in *T. armoricanus* were shown in red (clade 1), blue (clade 2), purple (clade 3) or green (clade 4) branch colors, respectively. Differentially expressed profile of cSP in *T. armoricanus* was shown by shades from green (down-regulated) to orange (up-regulated). The lay of the shades from innermost to outmost represented the relative fold changes in PH3d, OS3d, or OS15d, respectively. Tar, *T. armoricanus*; Pxy, *P. xylostella*; Dme, *D. melanogaster*. (**b**) RNA-seq and qPCR analysis of *cSP* family genes in *T. armoricanus*.

Sixty-eight *cSPs* in *T. armoricanus* were clustered into four clades (Figure 5a), one clade with Clip-domain serine protease homolog (*cSPH*), and other three clades with only *cSP*. Immune related cSPs, one *Gram-positive specific serine protease* (*Grass*) was clustered in clade 1, and *Persephone* (*Psh*) and *Spaetzle-processing enzyme* (*SPE*) were not identified in *T. armoricanus* genome. Meanwhile, developmental related cSP: *serine protease easter* (*EA*) and *serine protease snake* (*SNK*) were expanded in clade 1 and clade 2 (Figure 5a). Expression of *Grass* gene was not changed after two fungi infection, with very low expression (FPKM < 0.5). Although EA and SNK were involved in embryonic development clearly [51], 16 *EAs* and eight *SNKs* were differentially expressed after *P. hepiali* or *O. sinensis* infection (Figure 5a,b). Moreover, the serine protease without Clip-domain, Gastrulation-defectives (GD), which could interact with SNK and EA to form a proteolytic cascade [42], were also expanded in *T. armoricanus* genome (Figure S2a and Table S9), and 14 of which were differentially expressed after two fungi infection (Figure S2b). Among the 11 *Serpins* (*SPN*) identified in *T. armoricanus*, four were differentially expressed.

Forty-two key genes related to Toll pathway signal transduction differentially responded to three days after *P. hepiali* or *O. sinensis* infection, other four Toll pathway genes were specifically up-regulated in OS15d. Two *Tolls* (*TLs*) were up-regulated in PH3d, and one *TL* was only up-regulated in OS15d, indicating that *TL* genes might function in early infection of *P. hepiali* but in late infection of *O. sinensis*. *Serine/threonine-protein kinase pelle* (*Pelle*) was up-regulated after *P. hepiali* infection, but *myeloid differentiation primary response protein MyD88* (*MYD88*) and *Tube* were not responsive to *P. hepiali* or *O. sinensis* infection. Two *Rel/NF-κB* were identified in *T. armoricanus*: *Dorsal* (*DL*) and *Relish* (*REL*), but the *Dorsal-related immunity factor* (*DIF*) was missing. *DL*, *REL*, and *CACT* were up-regulated at day 3 either by *P. hepiali* or *O. sinensis* infection (Figure 4b and Table S10). In the IMD pathway, *death-related ced-3/NEDD2 like protein* (*DREDD*) were up-regulated after *P. hepiali* or *O. sinensis* infection, but the down-stream genes, *TAK1*, *TAB2*, *Kenny,* and *ird5* were not responsive. Most of the genes in the JNK signaling pathway did not respond to *P. hepiali* or *O. sinensis* infection (Table S10). After *P. hepiali* or *O. sinensis* infection, *UPD-1/2*, *Hopscotch,* and *STAM* were not responding. The inhibitors: *suppressor of cytokine signaling* (*SOCS*) and *cytokine-inducible SH2-containing protein* (*CIS*) of the JAK/STAT signaling pathway were up-regulated (Table S10), indicating that JNK and JAK/STAT signaling pathways played a limited role in defending against fungal infection. 

Microbial infection induces immune response effectors, which exhibit antimicrobial activity in insect hemolymph, and these immune response effectors are mainly composed of AMPs and LYZs [27]. In *T. armoricanus*, 25 AMPs were identified, including nine *ATTs*, four *DEFs* and four *CECs*, three *GLVs*, three *PRAMP1s*, and two *gallerimycins* (Table S9). Four *CECs*, three *ATTs* and one *GLV* were both up-regulated after *P. hepiali* or *O. sinensis* infection, indicating that these AMPs were involved in broad-spectrum antifungal processes in *T. armoricanus*. Fourteen AMPs including five *ATTs*, four *DEFs*, two *GLVs* and three *PRAMP1s* were specifically up-regulated at three days or 15 days after *O. sinensis* infection, whereas no AMPs were specifically up-regulated after *P. hepiali* infection. Meanwhile, 6 AMPs including 1 *DEF*, 2 *ATTs*, and 3 *PRAMP1s*, were specifically down-regulated after *P. hepiali* than that after *O. sinensis* infection (6 vs. 1) (Figure 4b,c and Figure S3d and Table S10), indicating that these AMPs might play key roles in defending against *O. sinensis* infection but not against *P. hepiali* infection. Additionally, 13 *LYZs* were identified, two of which were both up-regulated after *P. hepiali* or *O. sinensis* infection (Tables S9 and S10). 

From the above description, although the up-regulated genes in pathogen recognition and signaling modulation and transduction after *O. sinensis* were less than those following *P. hepiali*, more immune response effectors were up-regulated after *O. sinensis* infection but were suppressed after *P. hepiali* infection, establishing a foundation for the stronger resistance of *T. armoricanus* to *O. sinensis* infection.

### 3.7. PRAMP1s in Response to O. sinensis and P. hepiali infection

*PRAMP1* is an AMP with abundant proline residues, which responds in diverse ways in different insects against some fungi, such as *Pichia pastoris*, *Zygosaccharomyces marxianus*, and *Schizosaccharomyces pombe*, but not against *Aspergillus niger* and *Cryptococcus albidus* [48]. Three *PRAMP1* genes were identified in *T. armoricanus*, with a new potential motif (Figure S3a,b), and they were located nearby in the genome with similar CDS, intron, and untranslated region (UTR) lengths (Figure S3c), indicating that *PRAMP1* genes in *T. armoricanus* were tandem duplicated, probably generated by unequal crossing over. All three *PRAMP1* genes in *T. armoricanus* were significantly up-regulated at three days and 15 days after *O. sinensis* infection but were suppressed at three days after *P. hepiali* infection, as confirmed by qPCR (Figure S3d). It is likely that PRAMP1s in *T. armoricanus* are crucial for defending against early phase infection of *O. sinensis* as well as for maintaining the co-existence of *T. armoricanus* and *O. sinensis*, but did not contribute to *P. hepiali* defense.

### 3.8. Experimental Validation

**Figure 6:**
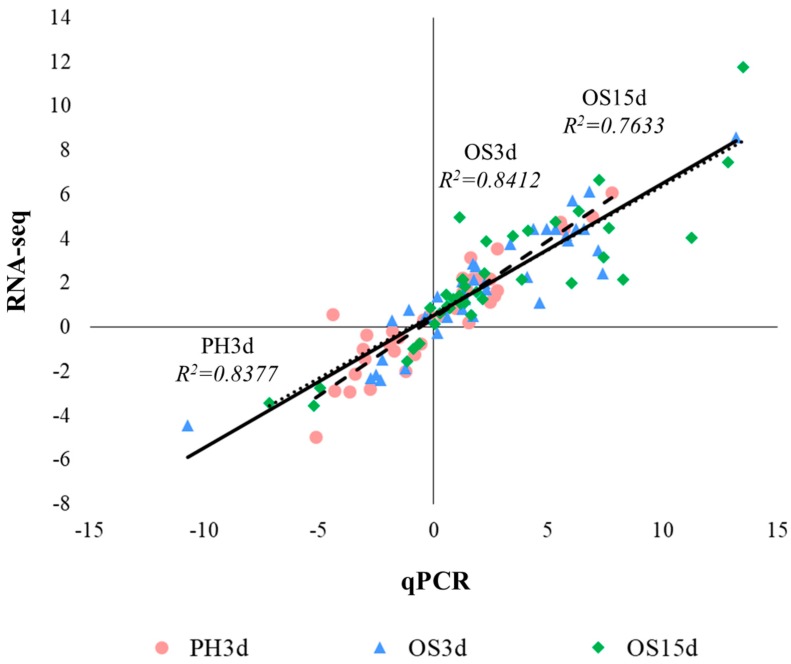
Validation of the selected DEGs in *T. armoricanus* from RNA-seq by qPCR. Comparison of the log_2_ of gene expression ratios between RNA-seq data and qPCR results. Cycle, square, triangle, and rhombus represent the results PH3d, OS3d, and OS15d, respectively. Correlation coefficient were also shown on the figure.

To validate the veracity and reliability of the DEGs identified by RNA-seq, 37 genes were selected for qPCR validation from those with differential expression patterns based on fold changes and functional enrichment results (Figure 4c and Figure 5b, Figure S3d and Figure S4). qPCR data significantly correlated with the RNA-seq result, with a correlation coefficient of 0.84 (for PH3d), 0.84 (for OS3d), and 0.76 (for OS15d) (Figure 6), demonstrating the credibility of the transcriptome results.

## 4. Discussion

After *P. hepiali* infection, *T. armoricanus* larvae usually survive no more than four days. In contrast, after *O. sinensis* infection, the larvae remain alive for several months before finally becoming mummified, without remarkable symptoms during the chronic infection. In this study, we suggested the potential mechanisms contributing to different responses of this insect to two fungi, by using RNA-seq analysis that was further confirmed by qPCR.

### 4.1. Common Responses in T. armoricanus After P. hepiali or O. sinensis Infection

**Figure 7:**
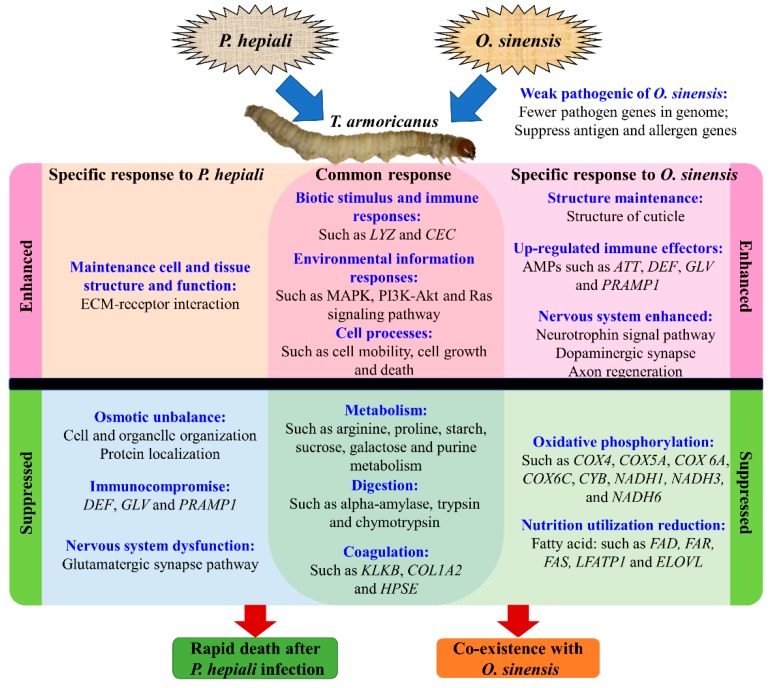
Overview of *T. armoricanus* larvae in response to *P. hepiali* or *O. sinensis* infection. Responses of *T. armoricanus* larvae after *P. hepiali* or *O. sinensis* infection were indicated. including the specific responses and common responses, the enhanced and suppressed responses were divided by black line.

Several common responses were found after *P. hepiali* and *O. sinensis* infection, including suppressed metabolic and coagulation pathways, and elevated genes expression related to biotic stimulus, immune response, environmental information responses and cell processes. Arginine, proline, starch, sucrose, galactose, and purine metabolism, digestion related genes such as *alpha-amylase*, *trypsin,* and *chymotrypsin* were suppressed in both PH3d and OS3d (Figure 7 and Table S11), indicating that reduction of these metabolic and digestion related genes expression was negative effect of fungal infection. Multiple coagulation related genes were also suppressed in both PH3d and OS3d (Figure 7), suggesting the susceptible coagulation system of *T. armoricanus* in response to fungi infection, and invalidation of the insect coagulation system may be an important common strategy by the entomopathogenic fungi.

In addition to the above down-regulated effects, three gene groups were involved in defending against two fungi (Figure 7). Firstly, the genes response to biotic stimulus and immune response were significantly up-regulated after infected by both fungi, including some specific immune effectors such as *CECs* and *LYZs*. Secondly, pathways involved in environmental information responses (such as MAPK and cAMP signaling pathway) were positive response to injected fungi. Thirdly, cell processes including motility, growth, and death, were also elevated after both fungi infection. Suggesting the important roles of these genes and pathways during fungal infection. 

Usually, entomopathogenic fungi can invade the host body by direct penetration of cuticle [28,52]. In *T. jiachaensis* larvae, cuticle and peritrophic membrane related genes were differentially expressed when the larvae infected (not injected) with *O. sinensis*. [52]. In the present study, approximately 77% cuticle genes were significantly differentially expressed in PH3d and OS3d, although the fungal spores were directly injected into the larval hemolymph, implying that the remodeling of insect cuticle genes may be an intrinsic response to the fungal infection, and is independent of the infection route.

The cSP family participates in immune responses and embryonic development, including hemolymph coagulation, melanotic encapsulation, induction of AMP synthesis, and activation of cytokines [53,54]. In *Drosophila*, immune related cSPs include *Grass*, *Psh,* and *SPE*. Toll pathway could be mediated by cascade formed by Modular serine protease (ModSP), Grass and SPE after fungi or gram-positive bacterium infection [55,56], or mediated by Psh and SPE that triggered by virulence factor independently of PRRs [57,58]. In our results, the *Psh*, *Grass,* or *SPE* was not identified in *T. armoricanus* genome or not expressed after *P. hepiali* or *O. sinensis* infection, suggesting the limited role of these known immune related cSP in *T. armoricanus* after these two fungi infection. Other cSP genes, *SNK* (homologue of *Psh*) and *EA* (homologue of *SPE*) are involved in embryonic development, which could interact with GD and Nudel to form a proteolytic cascade, culminating with proteolytic activation of SPZ and mediated the Toll pathway [59,60]. A recent study in shrimp *Penaeus monodon* show that the SNK-like could also activate the proPO system, which mediated the melanization in immune response [61], showing that the developmental-related cSP may also participate in immune response in different species. In our results, the *SNK*, *EA,* and *GD* were expanded in *T. armoricanus* genome, and multiple genes of *SNK*, *EA,* and *GD* were up-regulated after *P. hepiali* or *O. sinensis* infection, implying the proteolytic cascade formed by EA, SNK, GD, and Nudel might have multiple function in *T. armoricanus*. This proteolytic cascade might replace the functions of cascade formed by ModSP, Grass, and SPE, or formed by Psh and SPE in *T. armoricanus*, which is worthy of further research. Ghost moth is primitive lepidopteran species [27], and the *T. armoricanus* might have not evolved an immune-specific proteolytic cascade to participate in Toll pathway.

### 4.2. Rapid Death of T. armoricanus Larvae Infected by P. hepiali

Genes related to sterol hemostasis, osmotic balance, immune system, nervous system and food digestion in insect hosts are demonstrated to be five key targets by entomopathogenic fungi [62]. *P. hepiali* could kill *T. armoricanus* in four days, during *P. hepiali* infection, cell organization and protein localization were disordered, which might result in the disintegration and destruction of cell and organelles, and cause the destruction of osmotic balance. Immune effectors including *DEF*, *GLV*, *PRAMP1* were specifically down-regulated or not changed after *P. hepiali*, while up-regulated after *O. sinensis* infection (Figure 4 and Figure S3 and Table S11), suggesting that the mRNA expression of some specific AMPs in hemolymph was suppressed by *P. hepiali*. Entomopathogenic fungi can affect the nervous system by changing the serotonergic and dopamine synapse pathway. For example, the biting behavior in carpenter ants (*Camponotus castaneus*) is mediated by the change of dopamine pathway after *O. unilateralis* infection [63]. However, *P. hepiali* did not affect the serotonergic and dopamine synapse pathway in *T. armoricanus*, instead suppressed the glutamatergic synapse pathway (Figure 3), which is involved in locomotion and neuropathic pain [64,65]. Therefore, it appeared that three potential reasons were predicted for the rapid death of *T. armoricanus* larvae after *P. hepiali* infection: osmotic unbalance, immunocompromise, and nervous system dysfunction.

### 4.3. Co-Existence of O. sinensis and T. armoricanus

Compared to the rapid death of *T. armoricanus* larvae by *P. hepiali*, it would be interesting to know why *T. armoricanus* larvae could co-existence with *O. sinensis* over a long period. First of all, *T. armoricanus* larvae were probably associated with a weak pathogenic fungus, because fungal genome comparisons detected fewer pathogen-related genes in *O. sinensis* than in other entomopathogenic pathogens (such as *P. hepiali*, *Beauveria bassiana, Metarhizium robertsii*, etc.) [52,66], and the antigen and allergen genes of *O. sinensis* were decreased to protect the fungus to avoid from host immune responses [67]. Secondly, the specifically up-regulated genes in OS3d were more than those in PH3d, which were mainly involved in stimulus response, cellular process for macromolecules deliver and digest, and structural component. Thirdly, the expression of more AMPs such as *ATT*, *DEF*, *GLV*, and *PRAMP1* were specific increased after *O. sinensis* but decreased or unchanged after *P. hepiali* infection, the increase mRNA expression of these AMPs may contribute to prevent *T. armoricanus* from rapid death after *O. sinensis* infection. Moreover, neurotrophin signal pathway, dopaminergic synapse, and axon regeneration, was enhanced for locomotor activity, motivation, neural development, additional higher-order activities, and axon regenerate after injure. Finally, *T. armoricanus* larvae seemed to have adapted to the presence of *O. sinensis* after 15 days infection, most of the DEGs were restored to normal expression in OS15d. It was worth noting that six out of top 10 up-regulated genes in OS15d were unknown genes, without any structural or functional annotation (Table S11), suggesting that more research on the interactions or pathways associated with co-existence of *O. sinensis* and *T. armoricanus* is needed. In summary, besides the weak pathogenicity, it appeared that *O. sinensis* up-regulated more immune effector genes (such as AMPs) and pathways (cell and organelle structure, immunity system, nervous system, and stimulus response) in *T. armoricanus* larvae to defend the invaded fungi, preventing the rapid death of *T. armoricanus* and contributing to the co-existence of *T. armoricanus* and *O. sinensis*.

### 4.4. Conservation of the Insect and Fungus.

The previous study spanning nearly two decades and four countries (Bhutan, China, India, and Nepal) reveals that the Chinese cordyceps production is declining throughout much of the region, and its collapse would be due to both ecological (climate change) and social (overexploitation) reasons [68]. How to deal with the confliction between the increasing demand and the biological resource conservation is a major issue to be solved. The conservation of wild Chinese cordyceps has already attracted the attention of interactional public agencies, such as the World Wildlife Fund (WWF) and the Center for Agriculture Bioscience International (CABI). The Chinese Government also makes great effects to protect this biological resource by regulating the overexploitation [69]. Large-scale artificial cultivation of this Chinese cordyceps will alleviate the pressure of increasing demand and resource protection.

## 5. Conclusions

High-throughput RNA-seq with a reference genome provides opportunities for in-depth analysis of the ghost moth *T. armoricanus* in response to *P. hepiali* and *O. sinensis* infection. The results revealed the common and differential gene response caused by *P. hepiali* and *O. sinensis* and will help researchers to better understand the interaction mechanisms between this valuable insect and its associated fungi. The potential key genes for *T. armoricanus* response to *P. hepiali* and *O. sinensis* were discussed to improve the artificial production of this parasitic complex, complementing the study of interaction between insect hosts and entomopathogenic fungi.

## Figures and Tables

**Table 1:** Genes clusters of DEGs, based on the gene expression profiles in PH3d and OS3d.

Cluster	Number of DEGs	Expression Pattern		
		PH3d	OS3d	OS15d
Cluster 1	402	Down	Down	All Pattern
Cluster 2a	409	Down	Up/Unchanged	All Pattern
Cluster 2b	657	Up/Unchanged	Down	All Pattern
Cluster 3a	89	Unchanged	Unchanged	Down
Cluster 3b	185	Unchanged	Unchanged	Up
Cluster 4a	433	Up	Unchanged	All Pattern
Cluster 4b	765	Unchanged	Up	All Pattern
Cluster 5	166	Up	Up	All Pattern

**Table 2:** The differentially expressed immune or defense related genes in *T. armoricanus* larvae after *P. hepiali* or *O. sinensis* infection.

#	Immune Related Pathways	3 d						15 d	
		UP			Down			Up	Down
		C	PH	OS	C	PH	OS	OS	OS
1	Humoral immune response	18	50	23	5	10	14	42	8
	Pathogen recognition	3	19	5	3	2	5	7	5
	Signal modulation and transduction	5	29	4	1	2	8	12	2
	Toll pathway genes	4	26	4	1	2	7	9	2
	IMD pathway genes	1	1	0	0	0	1	3	0
	JAK/STAT pathway	0	2	0	0	0	0	0	0
	JNK pathway	0	0	0	0	0	0	0	0
	Pathogen elimination	10	2	14	1	6	1	23	1
2	Apoptosis	0	1	1	4	2	1	1	0
3	Autophagy	1	3	4	7	4	10	5	6
4	Coagulation	0	0	2	10	1	0	1	2
5	Encapsulation	1	0	0	3	1	0	0	0
6	Melanization	1	3	0	3	1	1	0	0
7	Phagocytosis	0	1	3	0	1	0	1	0
8	Removal of superoxide radicals	4	2	4	1	0	2	1	1
9	Small Regulatory RNA pathway	0	1	0	0	0	1	0	0
10	Others	0	5	6	1	3	4	2	1
	Total	25	66	43	31	23	33	53	18

3 d—3 days after infection; 15 d—15 days after infection; Up—up-regulated; Down—down-regulated; C—common genes in response to *P. hepiali* or *O. sinensis* infection; PH—infected with *P. hepiali*; OS—infected with *O. sinensis*.

## Supplementary Materials

The following are available online at https://www.mdpi.com/2075-4450/11/1/4/s1, Figure S1: Correlation and PCA analysis of sample replicates, Figure S2: GD family in *T. armoricanus*, Figure S3: *PRAMP1* genes in *T. armoricanus* with a potential new motif, Figure S4: qPCR analysis of the DEGs in *T. armoricanus* after *P. hepiali* or *O. sinensis* infection, Table S1: GO terms for predicting immune or defense related genes, Table S2: Primers for qPCR, Table S3: Statistic of clean read and HISAT2 alignment, Table S4: FPKM value of expressed genes in *T. armoricanus*. Table S5: Lists of DEGs in *T. armoricanus* larvae in response to *P. hepiali* or *O. sinensis* infection, Table S6: Expression tendency from three days to 15 days after *O. sinensis* infection, Table S7: GO comparison analysis between different clusters, Table S8: Pathways comparison analysis among different clusters, Table S9: Immunity or defense related genes in *T. armoricanus* genome, Table S10: Differentially expressed genes involved in immune or defense response in *T. armoricanus*, Table S11: The genes related to *P. hepiali* lethality and *O. sinensis* survival in *T. armoricanus*.
