# Supplementary figures and images for: Comparative Transcriptome Analysis of Thitarodes Armoricanus in Response to the Entomopathogenic Fungi Paecilomyces Hepiali and Ophiocordyceps Sinensis
Source: Insects. 2019 Dec 19;11(1):4. doi: 10.3390/insects11010004 (PMC7022891; doi:10.3390/insects11010004)

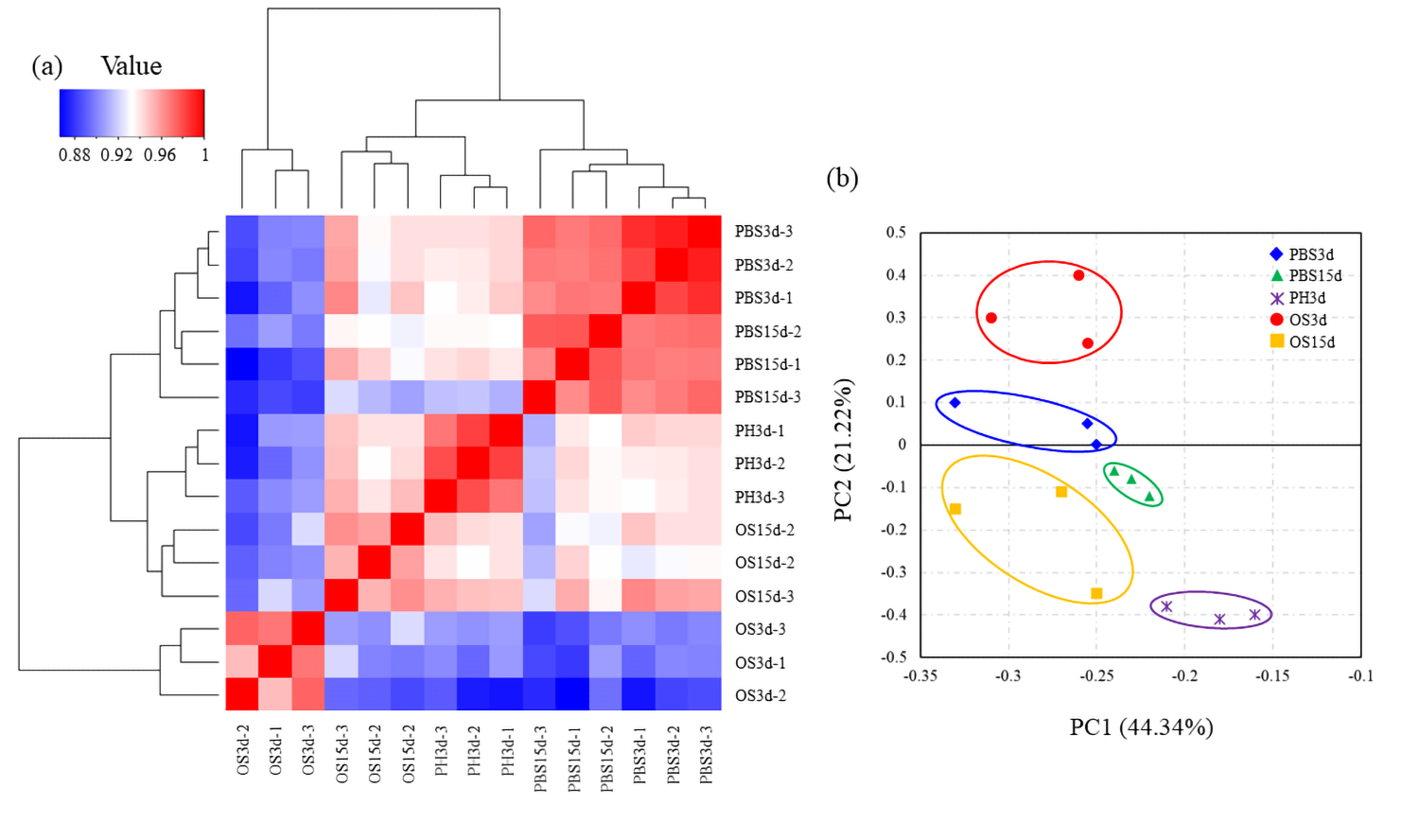

Supplement: Supplementary file 1 [file insects-11-00004-s001.zip › Figure S1.tif]

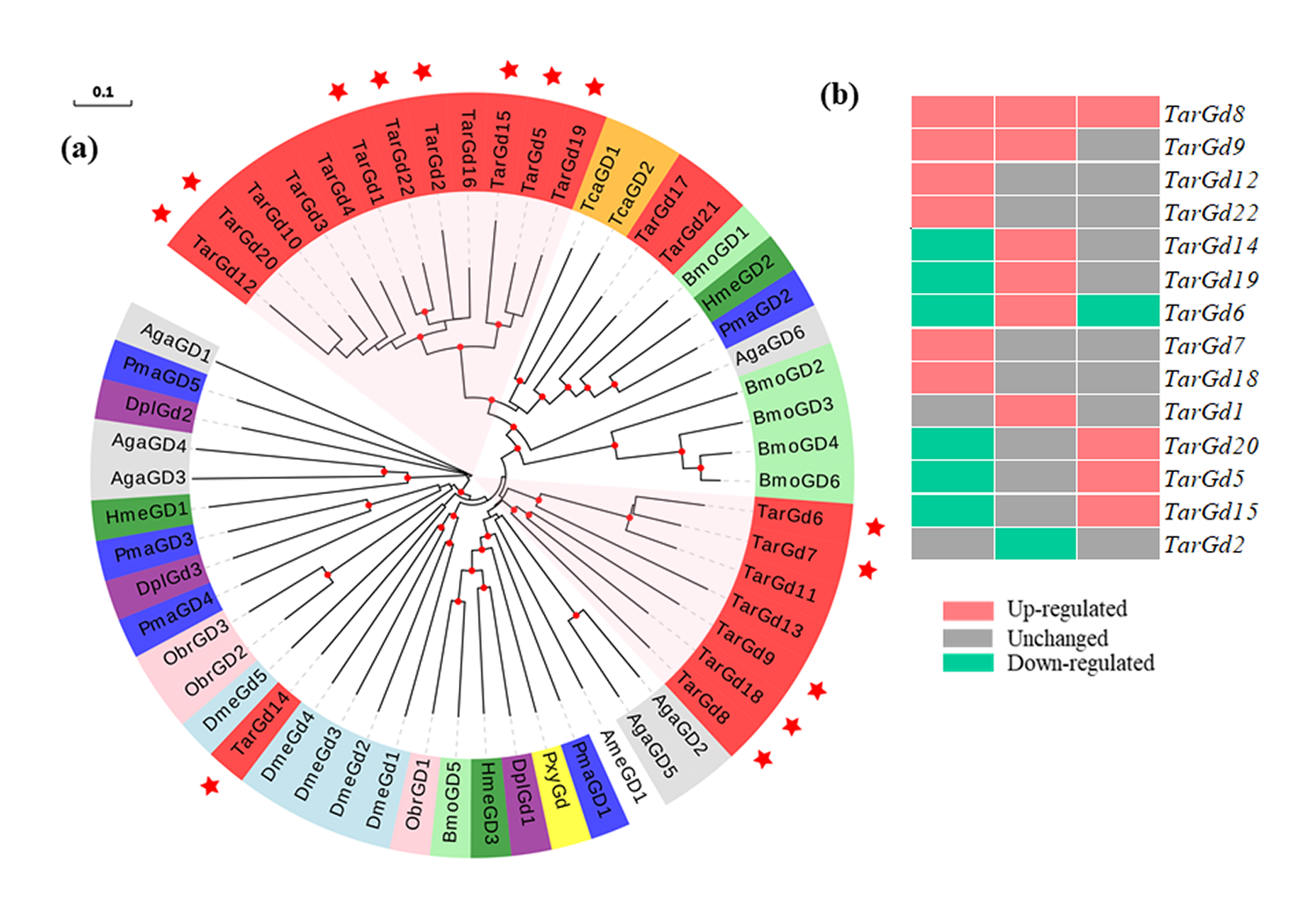

Supplement: Supplementary file 1 [file insects-11-00004-s001.zip › Figure S2.tif]

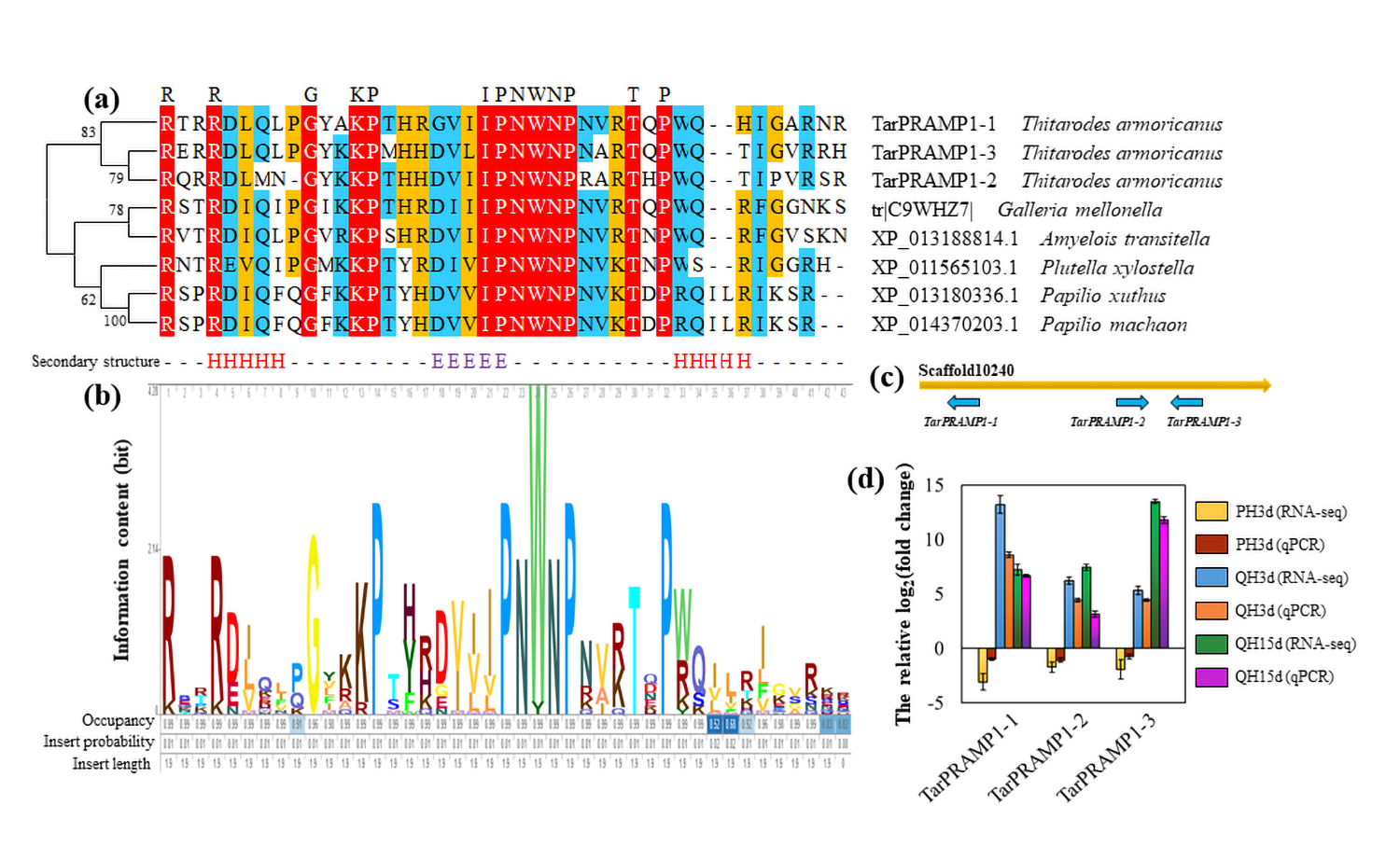

Supplement: Supplementary file 1 [file insects-11-00004-s001.zip › Figure S3.tif]

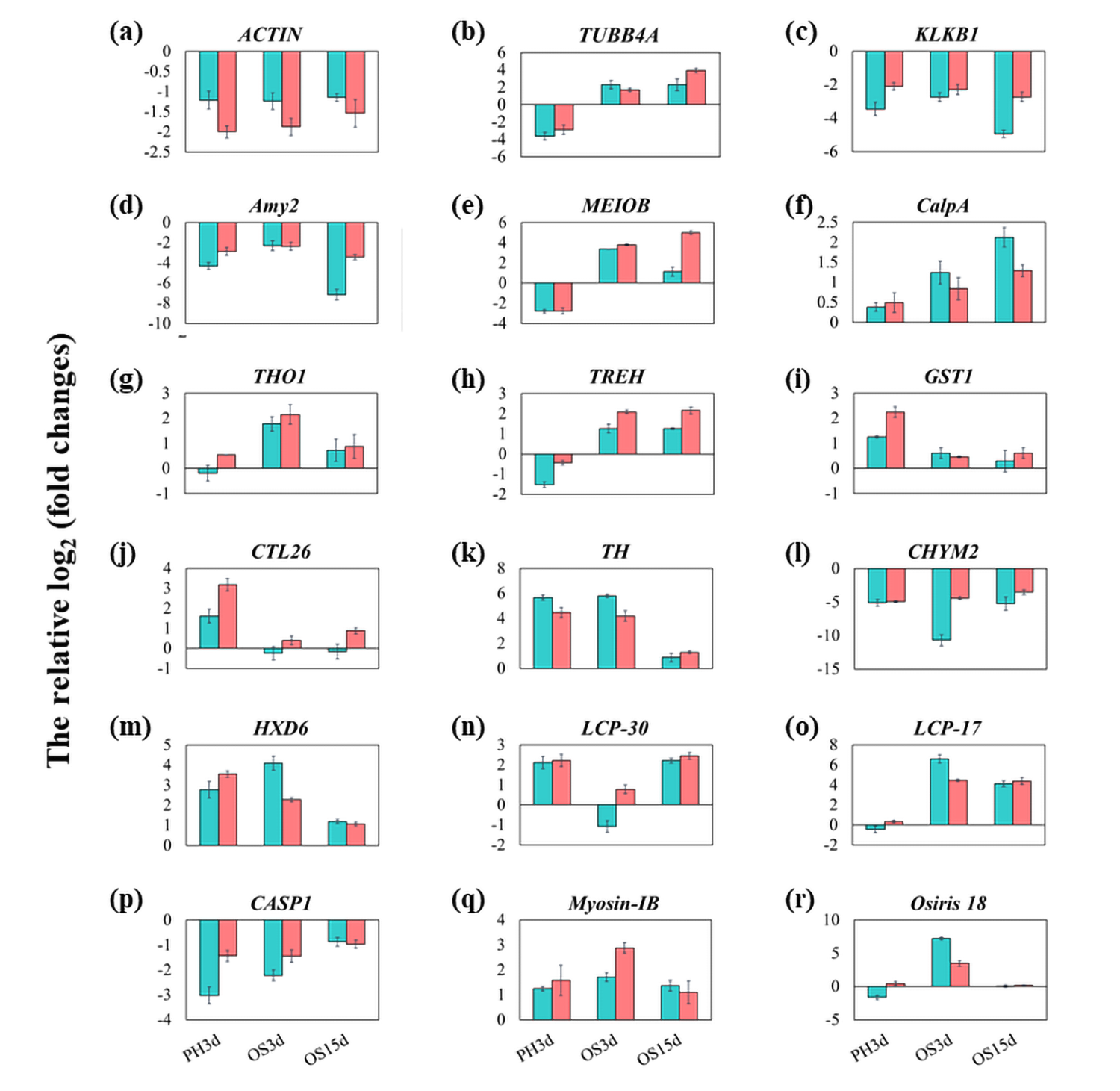

Supplement: Supplementary file 1 [file insects-11-00004-s001.zip › Figure S4.tif]
